# Identification and Characterisation of Trajectories of Sickness Absence Due to Musculoskeletal Pain: A 1-Year Population-based Study

**DOI:** 10.1007/s10926-022-10070-7

**Published:** 2022-09-14

**Authors:** Tarjei Rysstad, Margreth Grotle, Lene Aasdahl, Kate M. Dunn, Anne Therese Tveter

**Affiliations:** 1 Department of Physiotherapy, Faculty of Health Sciences, Oslo Metropolitan University, St. Olavs plass, P.O. Box 4, 0130 Oslo, Norway; 2 Research- and Communication Unit for Musculoskeletal Health, Oslo University Hospital, Oslo, Norway; 3 Department of Public Health and Nursing, Faculty of Medicine and Health Sciences, Norwegian University of Science and Technology, Trondheim, Norway; 4 Unicare Helsefort Rehabilitation Centre, Rissa, Norway; 5 School of Medicine, Keele University, Staffordshire, UK; 6 National Advisory Unit on Rehabilitation in Rheumatology, Diakonhjemmet Hospital, Oslo, Norway

**Keywords:** Sickness absence trajectory, Musculoskeletal disorders, Prognosis, Trajectories, Cohort studies

## Abstract

*Purpose* This study aimed to identify trajectories of sickness absence in workers on sick leave due to musculoskeletal disorders and explore the association between these trajectories and established prognostic factors for sickness absence. *Methods* We conducted a prospective cohort study of 549 workers (56% women, aged 18–67 years) on sick leave due to musculoskeletal disorders in Norway in 2018–2019. Sickness absence data were collected from the Norwegian sick leave registry and prognostic factors via self-reported baseline questionnaires. We used group-based trajectory modelling to define the different trajectories of sickness absence spanning a 1-year period. Multivariable multinomial logistic regression was used to estimate odds ratios and 95% confidence intervals for prognostic factors associated with the identified trajectory groups. *Results* We identified six distinct trajectories of sickness absence over 1 year: ‘fast decrease’ (27% of the cohort): ‘moderate decrease’ (22%); ‘slow decrease’ (12%); ‘u-shape’ (7%); ‘persistent moderate’ (13%); and ‘persistent high’ (18%). Prognostic factors, such as previous sickness absence days, return-to-work expectancy, workability, multisite pain, and health scores, differentiated between the sickness absence trajectories (all *P* < 0.05). Negative return-to-work expectancy was associated with the three trajectory groups with the highest number of sickness absence days (‘slow decrease’, ‘persistent moderate’, and ‘persistent high’). *Conclusions* This is the first study to explore the association of return-to-work expectancy with trajectories of sickness absence. Our findings highlight different patterns of sickness absence and the complex range of prognostic factors. These findings have implications for secondary and tertiary prevention strategies for work absence in workers with musculoskeletal disorders.

**Supplementary Information:**

The online version contains supplementary material available at 10.1007/s10926-022-10070-7.

## Introduction

Musculoskeletal disorders (MSDs) are the leading cause of sickness absence (SA) [1]. Trajectories of SA are complex, and include different states with different durations, often with recurrence [2, 3]. Traditionally, dichotomous outcomes of SA are explored cross-sectionally. However, measuring SA or return to work (RTW) at a single time point during follow-up ignores the complexity of SA. Understanding the SA patterns through longitudinal studies is clearly important to determining the best methods for preventing long-term SA in workers on sick leave with MSDs.

A growing body of research has found large heterogeneity in SA trajectories across several health conditions, which describes the recurring and fluctuating SA patterns over time [4, 5]. However, only a few studies have identified and characterised SA trajectories for workers on sick leave with a wide range of MSDs [3, 6–8]. Thus, the understanding of longitudinal patterns of SA in workers on sick leave with MSDs and how prognostic factors are related to these trajectories is incomplete [8]. Additionally, as most of these previous studies have used a retrospective design or self-reported sick leave data [3, 6, 7], which are described as suboptimal methods [9], studies with a prospective design using SA register-based data are warranted.

Several studies have reported different prognostic factors that have an impact on SA, such as age, gender, education level, and multisite pain [10, 11]. Recently, a synthesis of the evidence from systematic reviews found that expectations of RTW, ﻿pain, disability, and workplace factors are the most important modifiable factors in progressing SA across several disorders [11]. Although more than half of the included reviews addressed MSDs, they were primarily focussed on spine-related pain (neck and low back pain) [11]. However, there is strong substantive evidence that different MSDs share the same prognostic factors [12, 13], which highlights the need for more studies on identifying prognostic factors in study samples with a wide range of MSDs and not narrowing research to spine-related pain only.

By identifying individuals with similar trajectories of SA over time and exploring prognostic factors associated with these trajectories, it will be possible to provide more detailed information about future SA than using traditional approaches with dichotomous outcomes. Investigation of these issues is important in secondary and tertiary prevention efforts for work absence. Therefore, in this prospective cohort study, our aims were to identify distinct SA trajectories over a 1-year follow-up using longitudinal data from the National sick leave registry in Norway, and to explore the potential associations between these trajectories and established prognostic factors for SA.

## Methods

### Study Design and Sample

This prospective cohort study with a 1-year follow-up included individuals who received SA benefits from the Norwegian Labour and Welfare Service (NAV) between November 2018 and February 2019. Detailed information on the study design has been published elsewhere [14]. Briefly, all workers in Norway on sick leave for at least 4 weeks due to MSDs were sent an electronic invitation to participate. We included workers on sick leave due to a diagnosis within the musculoskeletal (L) chapter of the International Classification of Primary Care, second edition (ICPC-2) [15]. Participants had to be part of the working-age population in Norway (age 18–67 years). Individuals who had been on sick leave for less than 4 weeks were excluded because the probability of recovery during this period is high [16].﻿ Subjects were also excluded from enrolment in the study if they were unemployed or had insufficient Norwegian or English language skills. This study was reported in accordance with the Guidelines for Reporting on Latent Trajectory Studies (GRoLTS) checklist [17] and STROBE guidelines for reporting observational studies [18].

### Data Collection

All participants signed an electronic informed consent form and completed a web-based questionnaire. The date for completing the questionnaire was denoted as ‘first assessment’ for each participant. At first assessment, the median SA days in current spell was 35.8 days (interquartile range, 24.1–77.1). The questionnaire consisted of sociodemographic characteristics and prognostic factors associated with disability and work absence. Complete details regarding sociodemographic characteristics and questionnaires are provided elsewhere [14]. Longitudinal sick leave data were collected from the National Sick Leave Registry, which provided information about all registered medical benefits for each participant. Access to the sick leave register was granted by NAV in a secure form, and all data linkage was done according to Norwegian data law (NSD 861249).

We previously showed that the study sample is largely representative of the population of workers on sick leave due to MSDs in terms of the distribution of socio-demographic variables (age, gender, musculoskeletal diagnosis, occupational group, annual salary, and geographical location) [19].

### Sickness Absence

In Norway, all residents are included in the public insurance system. Medically certified sick leave can be graded from 20 to 100% and is compensated from day 1 up to 12 months. The first 16 days of the period of SA are paid by the employer and thereafter by the government. If the employer’s workability is still impaired after 12 months, it is possible to apply for work assessment allowance and disability pension, which both cover approximately 66% of the income. ﻿SA and disability pension can be received for full-time or part-time ordinary working hours, which means that the person can receive both part-time SA and disability pension concurrently.

The outcome of this study was the number of SA days per month of follow-up. We measured SA days as the number of lost workdays due to sick leave. To convert time on sick leave to actual time off work, we calculated SA days according to a 5-day workweek and adjusted for employment rate and the amount of sick leave. Using register data, no data on SA days were missing.

### Prognostic Factors of Sick Leave

The available dataset contained several candidate prognostic factors [14]. Prognostic factors considered important for the outcome were selected a priori based on previous evidence. Due to sample size consideration, we reduced the number of prognostic factors to a set of nine factors. A complete list of these prognostic factors is presented in Table 1, with measurement method, measurement unit, and missing values for each prognostic factor. The completion rate for the prognostic factors was greater than 98%. Missing data were considered missing at random (MAR); thus, all participants were included in the analyses. Due to the small proportion of missing data (< 2%) and little difference between responders and non-responders, a complete case analysis was performed.

**Table 1:** Characteristics of prognostic factors

Prognostic factor	Measurement unit	Measurement method	Data type	Missing observations, n (%)
Age	Years	Registry data	Cont	0
Gender	Male/female	Self-reported	Binary	0
Education level^a^	High/low	Self-reported	Binary	1 (0.2)
Sick leave days prior year	Days	Registry data	Cont	0
RTW expectancy^b^	0–10, 10 = worst	Self-reported	Cont	0
Workability^c^	0–10, 10 = best	Self-reported	Cont	1 (0.2)
Pain intensity^d^	0–10, 10 = worst	Self-reported	Cont	0
Multisite pain^e^	no = 0, yes = 1	Self-reported	Binary	0
Self-perceived health^f^	0–10, 10 = best	Self-reported	Cont	7 (1.3)

*Cont.* continuous, *RTW* return to work

^a^Grouped into two categories: low = no education, primary school and high school; high = higher vocational school or university

^b^Item 8 of the Örebro Musculoskeletal Pain Questionnaire Short Form [41]

^c^Single item from the Work Ability Index [42]

^d^Item 1 of the Keele STarT MSK tool [43]

^e^Item 5 of the Keele STarT MSK tool [43]

^f^Using EQ-VAS from the EQ-5D-5L. For a simpler interpretation of this factor, the scale was changed to 0–10 [44]

### Statistical Analysis

﻿Sociodemographic and candidate prognostic factors are presented as frequencies and percentages or means and standard deviations (SDs). To identify distinct trajectories of SA, we used group-based trajectory modelling (GBTM), which allowed us to estimate probabilities for multiple trajectories. We used the package *traj* for STATA (version 16.2) to determine trajectory groups of participants who followed a similar trajectory of SA over a 1-year window estimated by the highest probability that they belong in that group based on the maximum-likelihood method [20]. Accumulated days of SA was computed monthly from first assessment to 1-year follow-up for each participant. We treated days of SA as the dependent variable and time at each measuring point (month) as the independent variable. We used a censored normal model (Tobit model), which allows for clusters of data at the minimum or maximum of the scale. We fitted different models by applying and varying the number of groups from 1 to 6 and each group’s polynomial function (linear, quadratic, cubic, quartic, or quintic) [21].

To determine the most optimal model fit, we used a combination of different criteria: the Bayesian information criteria (BIC), the average posterior-probability for each group (minimum threshold of 0.7 indicates good internal reliability) [22], a minimum requirement of 5% group membership in the total sample size, and substantive interpretability of the model (i.e., distinct trajectory groups, narrow 95% confidence intervals, and clinical meaningfulness of the groups). We also estimated the relative entropy value to report on the performance of the classification (range 0–1, higher value indicates better class assignment) [17]. A Spaghetti plot was created to inspect the variability of individual trajectories within each group [23]. In addition, to ensure transparent reporting of the information concerning the number of cases allocated to each of the trajectory groups in each model, estimates for different models are presented in Supplementary Table 1.

Multivariable multinomial regression analyses using the least severe trajectory group as the reference were performed to examine the association between prognostic factors and the identified SA trajectory groups. All prognostic factors in the model were mutually adjusted. Prognostic factors recorded as continuous were kept continuous and not categorised to avoid loss of prognostic information [24]. One prognostic factor, level of education, was originally measured by four categories but grouped into two categories (low/high) to eliminate sparse categories and reduce the number of parameters in the regression model. Multicollinearity between independent variables was evaluated by tolerance, variance inflation factors (VIFs), eigenvalues, and the condition number. ﻿There was no evidence of multicollinearity between the variables in the model. To indicate the strength of associations, adjusted ORs with their 95% CIs were reported with *P*-values. We calculated Nagelkerke’s pseudo-*R*^2^ to estimate the overall model fit in a range of 0 to 1, where 1 indicates that all variation is explained. Moreover, to assess the size of the effect of each prognostic factor, we estimated the difference in *R*^2^ of the full model when the respective prognostic factor was removed [25].

All analyses were performed using STATA MP version 16.2 ﻿(StataCorp, College Station, TX, USA).

## Results

A total of 549 participants (median age 50.1 years, range 18.6–67.9; 56% women) were eligible for inclusion after excluding 160 who did not have a musculoskeletal diagnosis, 15 who were not on sick leave at first assessment, and 5 who had not been on sick leave the last 4 weeks before the first assessment.

### Sickness Absence Trajectories

**Fig. 1:**
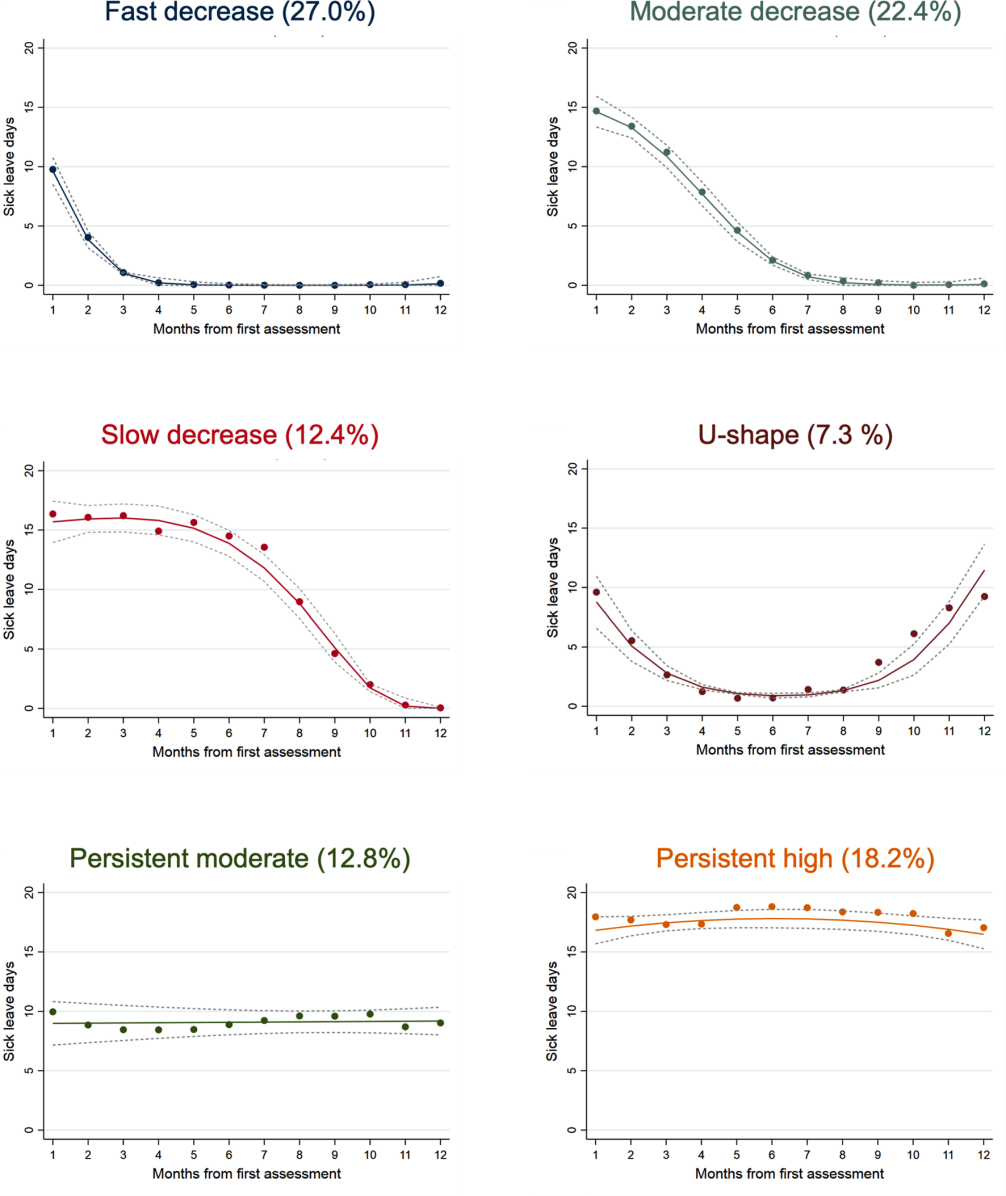
The six identified trajectories of sickness absence days among workers on sick leave due to musculoskeletal disorders over 1 year (N = 549). For each trajectory, the solid-coloured lines represent the predicted trajectory, and the grey dashed lines represent the 95% confidence intervals. The percentage of the cohort belonging to each trajectory group is above each figure

Group-based trajectory analyses identified six distinct trajectories based on SA days per month from first assessment (Fig. 1). This six-group model had readily interpretable and clinically relevant trajectories, with an entropy of 0.95, meaning that the model strongly separated the trajectories. The model had an adequate proportion and sample number in each group, all > 5% of the total sample size. For all groups, the average posterior probability was > 0.95, far above the recommended threshold of 0.7, indicating good reliability of the trajectories (Table S1). The spaghetti plots showed some variation of the individual trajectories, with the largest variation observed in trajectory groups five and six (Supplementary Fig. 1).

The first trajectory group, which we called ‘fast decrease’, was comprised of individuals who exhibited a fast decrease in SA days and an initial sustained first RTW. The largest proportion of the participants belonged to this group (27.0%). The ‘moderate decrease’ group (22.4%) and the ‘slow decrease’ group (12.4%) had a similar smooth shape as the first group but with a slower reduction in SA days. The fourth group had a pattern reflecting a fast decrease in the number of SA days within the first month, followed by a recurrence of work absence towards the end of the year, labelled the ‘u-shape’ group (7.3%). The fifth trajectory group represents the group of workers (12.8%) with a stable and moderate amount of SA days during the follow-up, labelled the ‘persistent moderate’ group. In the sixth trajectory, the ‘persistent high’ group, 100 (18.2%) workers had a stable and high amount of SA days throughout the 1-year follow-up.

### Participant Characteristics

The characteristics of the participants are provided in Table 2 according to each trajectory of SA. The median age ranged from 47.1 years in the ‘slow decrease’ group to 52.3 years in the group with ‘persistent high’ SA. Women were overrepresented in the ‘u-shaped’ group and ‘persistent moderate’ group. We also observed a clear difference in previous work absence, with a median of 30.0 SA days in the ‘fast decrease’ group compared to 80.4 SA days in the ‘high persisting’ group. Increases in disability pension were not present in the first three trajectory groups, whereas a small increase was observed in the ‘moderate persisting’ group (18.6%) and ‘high persisting’ group (6.0%). A higher number of SA days were observed in the ‘slow decrease’, ‘moderate persisting’, and ‘high persisting’ trajectory groups, with a median ranging from 120.3 to 221.1, far above the overall median of 67.1. Participants belonging to the remaining groups (‘fast decrease’, ‘moderate decrease’, and ‘u-shape’), had a median below the overall median. The trajectory groups with more SA days also reported higher pain intensity, higher degree of depressive symptoms, lower expectation of RTW, lower self-perceived health, and lower musculoskeletal health compared to the trajectory groups with fewer SA days. We also found substantially more participants in the ‘persistent high group’ wanting a new job. Higher degree of self-reported workability was observed in the ‘fast decrease’ and ‘u-shaped’ trajectories, with a mean value above the overall mean. We observed only modest differences in self-reported job satisfaction and work conflict across the six trajectory groups. Participants reporting pain duration > 1 year at first assessment were substantially overrepresented in the persisting groups (‘moderate persisting’ and ‘high persisting’).

**Table 2:** Characteristics of the cohort and across different sickness absence (SA) trajectory groups

	Overall	Trajectory 1	Trajectory 2	Trajectory 3	Trajectory 4	Trajectory 5	Trajectory 6
		Fast decrease SA	Moderate decrease SA	Slow decrease SA	U-shape SA	Persistent moderate SA	Persistent high SA
	n = 549	n = 148	n = 123	n = 68	n = 40	n = 70	n = 100
Median age, years (range)	50.1 (19–68)	49.2 (19–68)	48.6 (24–67)	47.1 (24–65)	50.1 (22–63)	50.8 (20–65)	52.3 (23–65)
Female	309 (56.3%)	75 (50.7%)	68 (55.3%)	33 (48.5%)	30 (75.0%)	52 (74.3%)	51 (51.0%)
Education level							
Lower education	40 (7.3%)	12 (8.1%)	6 (4.9%)	2 (2.9%)	4 (10.0%)	6 (8.6%)	10 (10.0%)
Higher education	288 (52.6%)	80 (54.1%)	60 (49.2%)	36 (52.9%)	14 (35.0%)	40 (57.1%)	58 (58.0%)
University (< 4 years)	131 (23.9%)	29 (19.6%)	31 (25.4%)	22 (32.4%)	15 (37.5%)	11 (15.7%)	23 (23.0%)
University (> 4 years)	89 (16.2%)	27 (18.2%)	25 (20.5%)	8 (11.8%)	7 (17.5%)	13 (18.6%)	9 (9.0%)
Norwegian as mother tongue	473 (86.5%)	130 (87.8%)	105 (86.1%)	58 (85.3%)	33 (82.5%)	63 (90.0%)	84 (84.9%)
Smoking	103 (18.9%)	27 (18.2%)	18 (14.9%)	11 (16.4%)	6 (15.4%)	22 (31.9%)	19 (18.8%)
SA days prior year^a^	37.8 (5.8–231.6)	30.0 (21.2–54.8)	35.0 (25.7–70.7)	36.7 (25.3–62.8)	37.1 (23.2–77.7)	45.1 (18.9–86.8)	80.4 (34.3–160.7)
SA days^a^	67.1 (26.4–133.2)	13.2 (5.9–22.9)	52.8 (39.8–70.7)	120.3 (87.6–152.1)	49.8 (30.0–62.7)	111.4 (89.7–127.1)	221.1 (187.2–241.4)
Increase in DP rate^b^	20 (3.6%)	0 (0.0%)	0 (0.0%)	0 (0.0%)	1 (2.5%)	13 (18.6%)	6 (6.0%)
Workability^c^	3.3 (2.7)	4.6 (2.7)	3.1 (2.7)	2.5 (2.3)	4.8 (2.2)	2.7 (2.0)	1.7 (2.2)
Pain intensity past week (0–10)^d^	6.3 (2.0)	5.9 (1.9)	6.2 (2.1)	6.7 (1.7)	5.8 (2.1)	6.5 (1.8)	6.9 (2.0)
Pain duration > 1 year	213 (38.8%)	46 (31.1%)	37 (30.1%)	22 (32.4%)	14 (35.0%)	40 (57.1%)	54 (54.0%)
Multisite pain	398 (72.5%)	94 (63.5%)	85 (69.1%)	49 (72.1%)	31 (77.5%)	61 (87.1%)	78 (78.0%)
RTW expectancy (0–10)^d,e^	3.6 (3.4)	1.8 (2.4)	2.7 (2.6)	4.0 (3.4)	2.1 (2.7)	5.0 (3.4)	6.7 (3.2)
Job satisfaction (0–10)^c^	7.5 (2.6)	7.6 (2.5)	7.8 (2.3)	7.7 (2.7)	7.9 (2.2)	7.4 (2.6)	6.9 (3.0)
Had a work conflict prior to sick leave	21 (3.9%)	5 (3.4%)	6 (5.0%)	2 (2.9%)	1 (2.5%)	2 (2.9%)	5 (5.0%)
Wants another job/position after sick leave	128 (23.6%)	31 (21.4%)	22 (18.0%)	17 (25.0%)	8 (20.0%)	14 (20.3%)	36 (36.4%)
Self-perceived health (0–10)^c^	5.2 (2.1)	6.3 (1.9)	5.1 (1.9)	4.8 (2.2)	5.5 (1.8)	4.7 (1.7)	4.2 (2.2)
Depressive symptoms (0–10)^d,e^	3.2 (3.0)	2.8 (2.9)	2.7 (2.9)	3.6 (2.9)	3.0 (2.7)	3.4 (2.9)	3.7 (3.2)
Musculoskeletal health (0–56)^f,c^	27.7 (8.2)	30.6 (8.0)	29.7 (7.6)	24.5 (7.6)	29.2 (7.6)	26.1 (6.3)	23.7 (8.7)

Values are given as n (%), median (IQR), or mean (SD) unless otherwise noted

*DP* disability pension, *IQR* interquartile range, *RTW* return to work, *SD* standard deviation

^a^Number of days on sick leave during the last 12 months before inclusion. Measured as calendar days and adjusted for partial sick leave and working rate

^b^Calculated as the increase in DP during the follow-up compared to baseline; e.g., if a participant had a 50% graded disability pension at baseline and 75% after 6 months, then a 25% increase has occurred

^c^A higher score is better

^d^Item from the ﻿Örebro Musculoskeletal Pain Screening Questionnaire Short Form [41]

^e^A lower score is better

^f^Measured with the Musculoskeletal Health Questionnaire (MSK-HQ) [45]

**Fig. 2:**
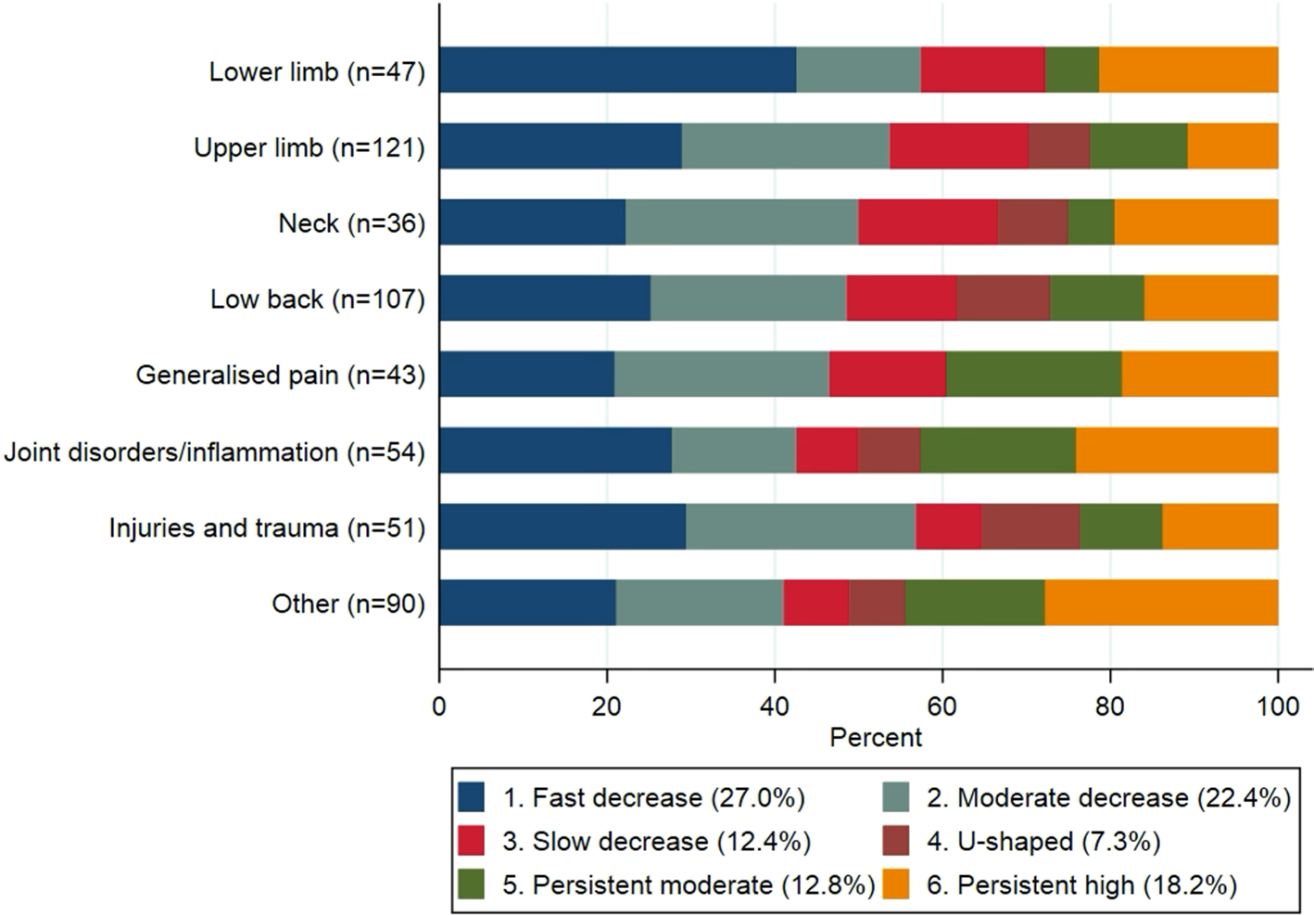
Distribution of the sickness absence trajectories according to musculoskeletal diagnosis (N = 549)

The distributions of the SA trajectories according to the musculoskeletal diagnoses leading to SA are shown in Fig. 2. Although the u-shaped trajectory was not present in the lower limb or generalised pain categories, the distribution of the SA trajectories did not differ substantially between the various musculoskeletal diagnoses.

### Prognostic Factors Associated with Sickness Absence Trajectories

The strongest adjusted associations, using the ‘fast decrease’ group as the reference category, are outlined in Table 3. Having lower RTW expectancy was associated with higher odds of belonging to the three trajectory groups with considerably more SA days (‘slow decrease’, ‘moderate persisting’, and ‘high persisting’). Having higher levels of self-perceived health (OR = 0.81, 95% CI 0.70–0.92) was associated with lower odds of belonging to the third trajectory group (i.e., ‘slow decreasing’), whereas being female (OR = 3.16, 95% CI 1.56–6.41) and having multisite pain (OR = 2.40, 95% CI 1.05–5.54) were associated with the ‘persistent moderate’ trajectory. Having more SA days in the prior year (OR = 1.02, 95% CI 1.01–1.03) was associated with higher odds of belonging to the ‘persistent high’ trajectory, whereas higher degrees of workability (OR = 0.70, 95% CI 0.60–0.82) decreased the odds of belonging to this trajectory. Age, education level, and pain intensity were not associated with any of the trajectories.

**Table 3:** Prognostic factors associated with each sickness absence (SA) trajectory using the ‘fast decrease’ trajectory group as the reference (N = 549)

	Trajectory 2	Trajectory 3	Trajectory 4	Trajectory 5	Trajectory 6	
	Moderate decreasing (n = 123)	Slow decreasing (n = 68)	U-shaped (n = 40)	Persistent moderate (n = 70)	Persistent high (n = 100)	Nagelkerke’s *R*^2^ difference^a^
Age	1.00 (0.98–1.02)	0.99 (0.96–1.02)	1.01 (0.98–1.06)	1.02 (0.98–1.05)	1.00(0.97–1.03)	0.004
Gender, women	1.13 (0.66–1.92)	0.87 (0.46–1.65)	**2.86 (1.23–6.65)**	3.16 (1.56–6.41)*	1.23 (0.63–2.41)	0.022
Education level, low	1.49 (0.87–2.57)	1.71 (0.89–3.30)	1.61 (0.76–3.45)	0.97 (0.49–1.92)	0.87 (0.42–1.78)	0.009
SA days prior year	1.01 (1.00–1.01)	1.00 (1.00–1.01)	1.01 (1.00–1.02)	1.00 (1.00–1.01)	1.02 (1.01–1.03)*	0.045
Negative RTW expectancy^b^	1.03 (0.92–1.15)	**1.18 (1.05–1.33)**	1.02 (0.86–1.20)	1.32 (1.16–1.49)*	1.39 (1.23–1.58)*	0.060
Workability^c^	0.83 (0.75–0.93)*	**0.81 (0.70–0.92)**	1.10 (0.93–1.29)	0.88 (0.77–1.02)	0.70 (0.60–0.82)*	0.040
Pain intensity^b^	1.04 (0.90–1.19)	1.13 (0.95–1.34)	0.98 (0.80–1.20)	0.98 (0.82–1.17)	1.15 (0.96–1.38)	0.005
Multisite pain^d^	1.15 (0.66–2.00)	1.18 (0.58–2.36)	1.84 (0.79–4.30)	**2.40 (1.05–5.54)**	1.31 (0.61–2.82)	0.007
Self-perceived health^c^	**0.81 (0.70–0.94)**	0.85 (0.71–1.01)	**0.77 (0.61–0.96)**	**0.81 (0.67–0.98)**	0.85 (0.71–1.02)	0.016

Values are given as adjusted odds ratio (95% confidence interval). Bold values indicate statistical significance at *P* < 0.05. *Indicates *P* < 0.001. Adjusted for all other variables listed in the table

*RTW* return to work

^a^Difference in Nagelkerke’s *R*^2^ between full model (*R*^2^ = 0.445) and model without the respective prognostic factor

^b^Measured continuously on a 0–10 scale, lower score is better

^c^Measured continuously on a 0–10 scale, higher score is better

^d^Measured dichotomously

The full model explained 45% of the variance between the trajectory groups (Nagelkerke’s *R*^2^ = 0.45). The prognostic factors with the greatest effect, estimated by the *R*^2^ difference, were RTW expectancy (*R*^2^ = 0.06), SA in the prior year (*R*^2^ = 0.05), and workability (*R*^2^ = 0.04; Table 3).

## Discussion

In this prospective cohort study of workers on sick leave due to MSDs, we identified six distinct trajectories of SA days over a 1-year follow-up and found that the distribution of participant characteristics varied across the trajectory groups. The trajectory group with the most SA days (‘high persistent’) comprised 18.2% of the participants, and we observed a small group (‘u-shape’, 7.3%) with early sustained RTW but a recurrence of work absence at the end of the year. We also found that the distribution of these trajectories did not vary substantially across different musculoskeletal diagnoses. Within three of the trajectory groups (‘slow decrease’, ‘moderate persisting’, and ‘high persisting’), a substantial number of SA days was observed, and RTW expectancy was associated with all these groups. These findings provide new insights into the complexity of SA in workers on sick leave with MSDs that propose important clinical and public health implications.

The trajectory groupings in our study are consistent with a recent Swedish study in individuals (N = 4894) with osteoarthritis, which found five SA trajectories: fast decrease, moderate fast, slow decrease, persistent high, and fluctuating [26]. Although this study is focussed on individuals with osteoarthritis, it is the most comparable study to ours regarding inclusion criteria, design, and follow-up time. Comparable SA trajectory groupings as in our study have also been identified in a recent large-scale sample of individuals with chronic non-cancer pain (N = 201,641) [27]. Other studies that included workers with a broader range of MSDs [6, 7, 28] have identified three to four SA trajectory groups. Explanations of the discrepancy in the number of trajectory groups compared to our study include different designs, methods, and samples. One of these studies included only female workers with MSDs in municipal kitchens [28], and all studies included people who were not already on sick leave at baseline, resulting in most of the cohort (59%–76%) belonging in a trajectory with no/almost no SA days. Another study in workers with MSDs found 2132 unique trajectories with nine clusters [8]. However, this study used a different modelling method (sequence analysis), making it difficult to directly compare it to our results.

Our findings regarding different characteristics between all trajectory groups corroborate previous studies. Trajectory groups characterised by a high number of SA days tended to be overrepresented by women [2, 6, 26, 29], individuals with more days on sick leave the previous year [26, 29], and individuals who tended to score higher on pain intensity [3, 6], multisite pain [2, 6, 7, 28], and depressive symptoms [3, 28]. Although some studies have reported higher proportions of smokers in high SA trajectories [2, 6, 28], we were only able to identify this in the ‘moderate persisting’ trajectory. In our study, the proportion of workers that did not have Norwegian as their mother tongue was stable between the trajectory groups. This contrasts Swedish studies [26, 29], which found that trajectories with high SA days had more individuals born outside Sweden and the EU.

Although a recent trajectory study by McLeod et al. [8] pointed out the necessity to differentiate between specific diagnoses when investigating trajectories, we were unable to identify that SA trajectories varied across different musculoskeletal diagnoses. This discrepancy may be explained by the lack of specific diagnoses in our study. However, our observation is in accordance with previous studies and reviews that showed a similarity in prognosis regardless of musculoskeletal diagnosis [12, 13]. These findings indicate that prognosis may be more important than diagnosis, as recently argued by Croft et al. [30].

Negative expectancy of RTW was a prognostic factor for SA, with an OR ranging from 1.18 to 1.39 for the three trajectory groups with the greatest number of SA days. According to this model, relative to the reference group, the odds of having persistently high SA throughout a year increases 39% per 1 unit increase in negative RTW expectancy. Our results add to previous evidence highlighting that expectancy of RTW is an important prognostic factor of SA [31, 32], and corresponds to research into trajectories of pain, where the patients’ recovery expectations have shown to be an important predictor [33–35]. This finding highlights the importance of expectancy as a potentially modifiable factor whatever the outcome is, which has also been highlighted in a recent Cochrane review [36]. Yet, to the best of our knowledge, our study is the first to explore the association of RTW expectancy with trajectories of SA.

We also confirmed other well-known prognostic factors associated with increased SA days; low workability, multisite pain, and poor general health were associated with trajectories with an increased number of SA days. This observation is in accordance with previous trajectory studies [2, 7, 28]. The highest significant ORs were observed for female gender (OR = 2.86, 95% CI 1.23–6.65) in the ‘u-shape’ group and female gender (OR = 3.16, 95% CI 1.56–6.41) in the ‘persistent moderate’ group. However, the wide CIs for these estimates indicate great uncertainty about the true OR and prognostic effect of these factors. Previous studies reported conflicting results for female gender as a predictor of SA [37], whereas other studies have found it to be an important prognostic factor for predicting poor SA outcomes and trajectories [11, 27]. Multisite pain was found to be associated with persistently moderate SA (OR = 2.40, 95% CI 1.05–5.54). Although the wide CI indicated some vagueness about this relationship, this finding is in agreement with earlier observations in which multisite pain seemed more important than pain intensity in predicting SA [6].

Previous trajectory research has found many prognostic factors that are associated with SA and RTW but often with conflicting and diverse results. The reason for the differences is not fully known, but a number of factors may contribute, such as the different trajectory groups of SA. Combined with the results of previous studies, our findings confirm that different trajectory groups are associated with different prognostic factors. Another explanation for this diversity may be the different methods used to investigate prognostic factors. Though some studies have reported adjusted results, other studies have reported unadjusted results or a combination of both. Studies that provide unadjusted estimates are the least conclusive because they are not adjusted for important and known covariates, which may overstate their conclusions [36, 38]. Therefore, in our study, we conducted confirmatory analyses of prognostic factors, planned a priori, to explore the association between these factors and the different trajectories of SA controlled for other important prognostic factors.

As work absence often involves repetitive and recurrent absence periods of varying duration, analysis of SA is challenging. Using group-based trajectory modelling, our study yields insights into the longitudinal complexity of SA that could not be found using conventional logistic or Cox regression analysis. Moreover, given the large number of SA days in half of the trajectories, early detection and new treatment strategies considering some of these modifiable prognostic factors are important in preventing long-term work absence. One way to enhance this is by using trajectory subgrouping, which can allow a more subtle and precise classification of workers on sick leave who are at high risk of work absence [39]. This could be explored in future studies.

### Strengths and Limitations

The main strengths of the present study are the longitudinal prospective design, prospective data collection, representative sample, low volume of missing data for the prognostic factors, and use of comprehensive register-based data, which enabled us to have repeated measures of outcome data for each participant with no missing data, eliminating recall bias. Moreover, we think our study has good face validity because it has been conducted in a social insurance setting where all workers on sick leave in Norway are contacted and followed up.

The following limitations need to be considered when interpreting this study. First, we found trajectory groups with good internal reliability, but these trajectories need to be externally validated in separate samples of workers on sick leave due to MSDs. Although Nagin and Odgers [22] cautioned against the mission to identify the true number of trajectories, replicating findings is essential in prognosis [24] and trajectory research [39]. Second, the small sample size in the smallest groups leads to some uncertainty regarding the regression analysis. We tried to mitigate this risk a priori by selecting nine prognostic factors based on three to four expected trajectories, but we ended up identifying more trajectories, resulting in a small sample size in some of the groups. Therefore, we cannot firmly establish the prognostic relationships, though our multivariable model was adjusted with the most relevant covariates that may have affected these associations. Third, although RTW expectation was the most important prognostic factor in our study, the question also includes home duties, which may have reduced the prognostic information on work-related expectancy. Future studies should include expectation questions that emphasise RTW or SA only. Fourth, as we left-censored our data from the first assessment rather than from the index date of the current spell for each participant, the number of SA days prior to the trajectory start differs. This was a pragmatic decision, mainly driven by the timing of the data collection in our cohort and the fact that we wanted a similar starting point when exploring the associations between prognostic factors and trajectory groups. However, this needs to be considered when comparing our results to other studies. Fifth, a different trajectory method (e.g., latent class growth analysis [LCGA]) may have resulted in different trajectory groupings and shapes [40]. However, a recent methodological study compared GBTM as used here to LCGA with work absence data and showed only small differences between the two methods in terms of groupings and shapes [23]. Finally, due to the variation in legislation on SA across different countries, the comparability of our findings may be restricted to countries with similar benefit systems, such as other Nordic countries.

## Conclusion

Using trajectory modelling in a representative sample of workers on sick leave with MSDs, we found that nearly half of the sample had trajectories with a high amount of SA. We found that those with a persistently high SA pattern seemed to reflect a continuation of the previous sick leave. This pattern was also associated with lower RTW expectancy and workability. We also observed that prognostic factors seemed to differ across the various trajectories. These findings show that individuals who had an MSD-related SA experienced a complex and heterogenic process of returning to work, and that a sustained RTW may still lead to recurrence of work absence many months later.

## Supplementary Information

Below is the link to the electronic supplementary material.

Supplementary file1 (PDF 163 kb)

## Data Availability

The datasets used and/or analysed during the current study are available from the corresponding author on reasonable request.
