# Supplementary material for: Identification and Characterisation of Trajectories of Sickness Absence Due to Musculoskeletal Pain: A 1-Year Population-based Study
Source: J Occup Rehabil. 2022 Sep 14;33(2):277–87. doi: 10.1007/s10926-022-10070-7 (PMC10172278; doi:10.1007/s10926-022-10070-7)
Supplement: Supplementary file 1 — Supplementary file1 (PDF 163 kb) [file 10926_2022_10070_MOESM1_ESM.pdf]

SUPPLEMENTARY INFORMATION

Supplementary Table 1. Model selection results.

No. of groups	Trajectory shapes[a]	BIC (N=549)	SSABIC (N=13725)	AIC	Trajectory group proportion (%)[b]	Average posterior probability	Entropy
1	2	-16775.11	-16780.08	-16766.49	100	.	.
2	2 2	-14819.02	-14828.96	-14801.78	58.2/41.8	0.99/0.99	0.959
3	2 2 2	-14334.96	-14349.87	-14309.11	42.2/23.4/34.4	0.98/0.96/0.99	0.953
4	2 2 2 2	-13908.20	-13928.08	-13873.74	39.7/16.3/21.0/23.0	0.99/0.98/0.98/0.96	0.955
5	2 2 2 2 2	-13715.21	-13740.06	-13672.13	20.1/29.7/14.9/16.1/19.3	0.95/0.95/0.97/0.97/0.99	0.929
6	2 2 2 2 2 2	-13557.99	-13567.80	-13486.29	16.7/18.1/22.6/8.6/15.1/18.8	0.94/0.95/0.93/0.97/0.98	0.903
6[c]	2 2 1 2 3 3	-13492.88	-13523.94	-13439.03	27.0/7.3/12.8/18.2/22.4/12.4	0.98/0.96/0.96/0.97/0.97	0.951

Abbreviations: AIC, Akaike information Criterion; BIC, Bayesian Information Criterion; SSABIC, Sample Size Adjusted Bayesian Information Criterion.

[a]Trajectory shapes: 0=intercept, 1=linear, 2=quadratic, 3=cubic.

[b]The proportion in each group based on the assignments for the maximum posterior probability.

[c]This model demonstrated the lowest BIC and the best clinical relevance, while conserving excellent average posterior probability and entropy.

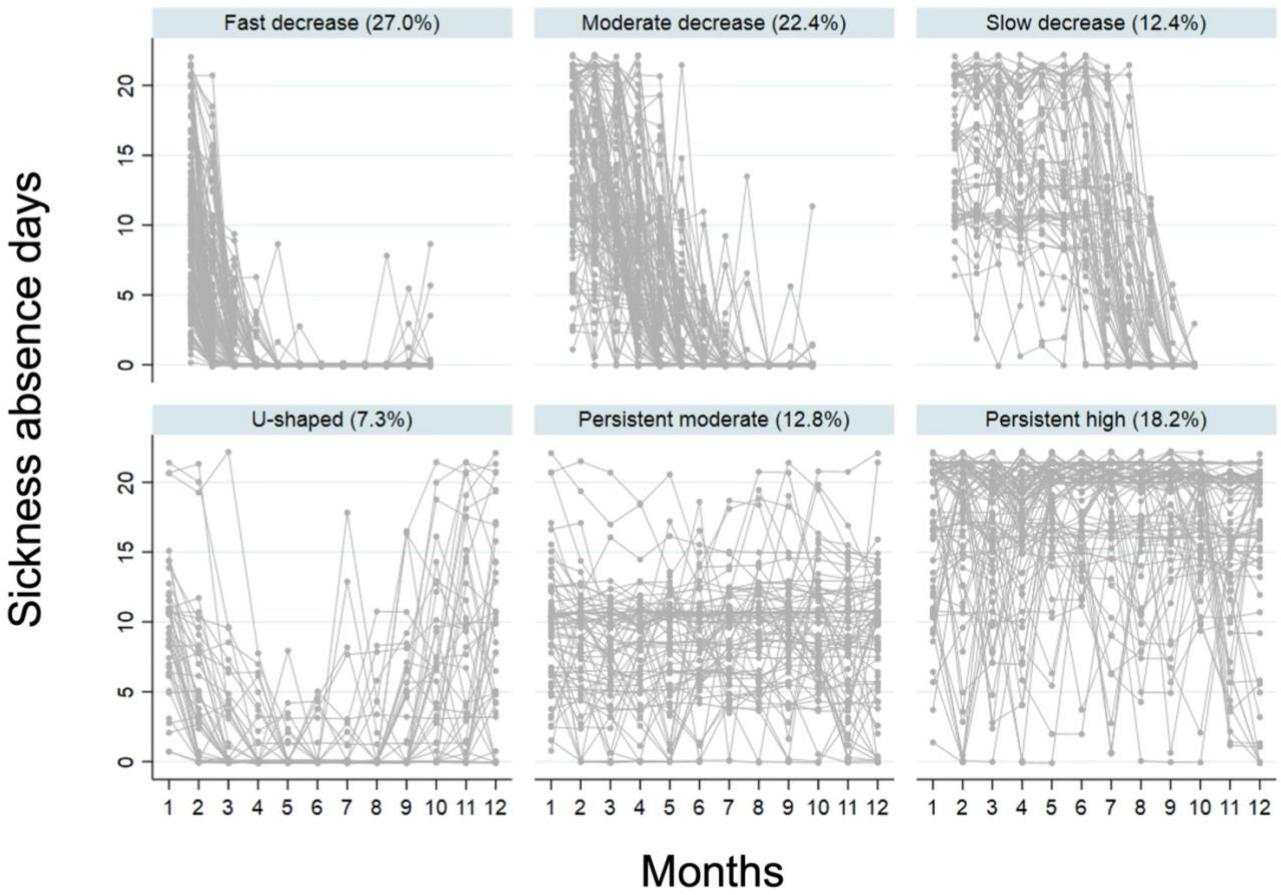

Supplementary Figure 1. Spaghetti plots over days on sick leave within each trajectory group among a representative sample of workers on sick leave due to musculoskeletal disorders (N = 549).
